# On the Treatment of Inflamed Breasts

**Published:** 1855-11

**Authors:** M. Reitzenbeck

**Affiliations:** Prague


*On the Treatment of Inflamed Breasts of Nurses.* By M. REITZENBECK, of Prague.

The method here recommended is so simple, that no one need hesitate to adopt it, provided he is called in before the mischief has reached a certain degree of development.

It is well known that engorgements of the mammary glands are frequently caused by chapped nipple. The inflammation of the skin extends directly into the ducts, exudations take place by which some of these ducts are plugged up, the milk is pent in, and hence the engorgement. If now, in such a case, the breast be surrounded with the hands, and pressure made in the direction of the nipple, a thin, transparent, whitish vesicle, is caused, by the milk accumulating behind the closed orifices of the ducts. It is necessary then, to do this, and having done it, the next thing is to prick the vesicle with a needle, to remove any epithelial scales which may be present, and to apply the infant. If time has not been lost unnecessarily, the relief is most immediate, and pain and tumefaction disappear in a few minutes; but even when it is otherwise the relief is very marked, and by repeating the process a few times, the sufferer is relieved altogether.—*Gaz. Medicale de Paris*, and *Ibid.*

